# Hematological manifestations in patients newly diagnosed with pulmonary tuberculosis

**DOI:** 10.12669/pjms.38.7.5911

**Published:** 2022

**Authors:** Yasmeen Batool, Gulzaib Pervaiz, Amna Arooj, Sabeen Fatima

**Affiliations:** 1 Dr. Yasmeen Batool, MBBS, FCPS (Hematology). Assistant Professor Pathology, Pathology Department, Nishtar Medical University & Hospital, Multan, Pakistan; 2 Dr. Gulzaib Pervaiz, MBBS. Demonstrator, Community Medicine Department, Rawalpindi Medical University, Rawalpindi, Pakistan; 3 Dr. Amna Arooj, MBBS, FCPS (Hematology). Assistant Professor Pathology, Pathology Department, Sahiwal Medical College, Sahiwal, Pakistan; 4 Dr. Sabeen Fatima, MBBS, MPhil (Hematology). Women Medical Officer, Pathology Department, Nishtar Medical University & Hospital, Multan, Pakistan

**Keywords:** Anemia, Leukocytosis, Pulmonary Tuberculosis, Thrombocytosis, Leucopenia

## Abstract

**Objective:**

To determine the frequency of various hematological manifestations among newly diagnosed cases of pulmonary tuberculosis on complete blood counts.

**Methods:**

This retrospective cross-sectional study was conducted on 500 newly diagnosed patients of pulmonary tuberculosis in hematology department of Nishtar Medical University from 1^st^ November 2020 to 30^th^ April 2021 using consecutive sampling. Detailed history and complete general physical examination findings, complete blood picture including erythrocyte sedimentation rate were retrieved from hospital electronic medical records system. Data was collected on a specially designed Proforma, entered into SPSS version 23.0 and analyzed. Data is presented as mean±SD and frequency and percentages for numerical and categorical variables respectively using tables and figures.

**Results:**

In this study the mean age of patients was 34.36 ± 6.41 years with male to female ratio 2.52: 1. Frequency of anemia was 82.6% (n=413), leukocytosis was 46.2% (n=231) and leucopenia in 102 patients (20.4%). Thrombocytosis was detected in 131(26.2%) patients. Raised ESR was observed in 495(99%) of the patients. No association of age and gender was observed with hematological manifestations. However, thrombocytosis was significantly more common in male patients (p-value = 0. 008).

**Conclusion:**

Hematological parameters like high Erythrocyte Sedimentation Rate (ESR) and anemia were commonly detected in newly diagnosed patients with pulmonary tuberculosis. Thrombocytosis was seen more commonly in male patients of pulmonary tuberculosis.

## INTRODUCTION

Tuberculosis (TB) is a common and often deadly infectious disease caused by mycobacterium usually Mycobacterium tuberculosis in humans. *Mycobacterium tuberculosis* is an acid fast facultative intracellular rod shaped obligate aerobe which prefers especially to localize in macrophages.^1^ TB is the world’s second most common cause of death from infectious disease, after HIV/AIDS.^2^ An estimated 510,000 people including around 15000 children acquire TB infection in Pakistan that leads to more than 70,000 deaths due to the single deadly infection in the country annually.^3^ The spread of this disease is caused by several factors notably the HIV/AIDS epidemic, low socio-economic status, overcrowding and malnutrition.^4^-^6^ A study in Karachi provides evidence for a strong positive association between tuberculosis and diabetes.^7^

A study in Jamshoro detected primary multi drug resistant tuberculosis more commonly in previously diagnosed and treated patients but also in newly diagnosed pulmonary patients and its due to dissemination of MDR cases within the community, difficult to treat.^8^ TB is a curable disease of high morbidity, causing a great economic burden on health services.^9^ Establishing the diagnosis of TB in Pakistan is still a dilemma where a majority of the patients are poor and cannot afford expensive diagnostic tests like polymerase chain (PCR), QuantiFERON-TB gold and gene expert tests.^9^-^11^ Hematopoietic system is also seriously affected by tuberculosis. It exerts a dazzling variety of hematological effects involving both cell lines and plasma components.^12^

In an endemic area like Multan, no such study has been conducted with special emphasis on hematological changes in TB. Hematological changes in tuberculosis act as a hallmark of disease and help in early diagnosis, assessing the prognosis as well as assessing therapeutic response. Thus, the rationale is knowing the magnitude of hematological aberrations that may be regarded useful marker of disease activity, mortality risk and monitoring therapy. It will prevent unnecessary investigations and will result in better management of Pulmonary TB patients.

The study aims to determine the frequency of various hematological manifestations among newly diagnosed cases of pulmonary tuberculosis on peripheral blood picture and ESR.

## METHODS

This retrospective cross-sectional study was conducted on 500 newly diagnosed patients of pulmonary tuberculosis in hematology department of Nishtar Medical University from 1^st^ November 2020 to 30^th^ April 2021 using consecutive sampling. The study was conducted after permission from the ethics review committee (ERC No -18674 Sept. 25^th^ 2021). Clinical, demographic and lab data of patients of either gender being newly diagnosed cases of Pulmonary tuberculosis, between ages 25-45 years, attending or admitted through OPD/Indoor of Nishtar Hospital Multan was retrieved from electronic medical records system by consecutively selecting their medical registration numbers. A new case of pulmonary tuberculosis was defined as a patient who has never received treatment for tuberculosis or who had taken anti tubercular therapy (ATT) for less than one month. Results of complete blood counts (CBC) and ESR at the time of diagnosis were included in the study. Hematological manifestations were defined as anemia (hemoglobin < 12 mg/dL in females and <14 mg/dL in males), Leukocytosis as (leukocyte count > 11000 /mm^3)^, leucopenia (leukocyte count<4000 /mm^3)^, thrombocytosis (platelet’s count >450 /mm^3)^, thrombocytopenia (platelets count <150 /mm^3^) and raised ESR(in males above 15mm/hr) and in females above 20 mm/hr). To exclude patients already diagnosed of hematological disorders like bone marrow failure ,immune thrombocytopenia, leukemia ,lymphoma and patients receiving chemotherapy, with other lung pathology or patients already receiving anti-tuberculosis drugs for a month or more than a month , on medical records. Indoor patients’ medical chart history was evaluated and outdoor patients were contacted on the mobile phone.

All the information was collected on a specially designed Proforma. All the collected data were entered into SPSS version 23.0 and analyzed. The qualitative data like demographics, anemia, leukocytosis, leucopenia, thrombocytosis, thrombocytopenia and raised ESR were presented as frequency distribution. Quantitative data like age in years and Hb%, TLC, platelets and ESR were presented as means ± standard deviations. Data were stratified on age and gender to deal with effect modifiers. Post-stratification Chi-square test was applied. P-value ≤ 0.05 is considered significant.

**Table-I:** Demographic Characteristics of study participants (N=500)

Characteristics		Frequency (n)	Percentage (%)
Age Groups (years)	20-29	156	31.2
	30-39	204	40.8
	>39	140	28.0
Gender	Male	358	71.0
	Female	142	28.

## RESULTS

The study included 500 patients, with the mean age being 34.36 ± 6.4. The most common age group was 30-39 years followed by 20-29 years and > 39 years of age. There were 358 (71.6%) males and 142 (28.4%) females’ patients in present study with male to female ratio 2.52: 1.

**Fig.1:**
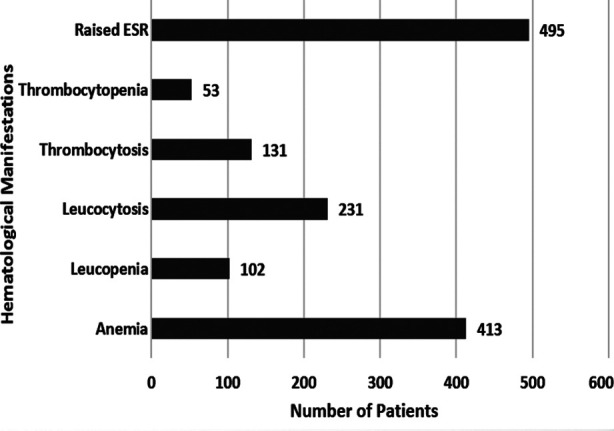
Hematological Manifestations in patients newly diagnosed with Pulmonary Tuberculosis (N=500).

## DISCUSSION

Reversible peripheral blood abnormalities are commonly associated with pulmonary tuberculosis. Insight into the relationship between haematological abnormalities and mycobacterial infection has come from an understanding of the immunology of mycobacterial infection.^5^ In our study that showed male predominance, anemia was seen in 82.6% of patients. Similar finding were noted by Erhabor where anemia was seen in 88.7% of cases with male predominance.^13^ In contrast to it, Shafee in Quetta noted female predominance and anemia was seen in 55% males and in 53% females.^12^ In India, Mukherjee reported anemia in 72.7% of patients and its severity had significant associations with body mass index that was an independent predictor of anemia.^14^

In contrast to it, Kahase reported a lower frequency of anemia as 25% of subjects were anemic.^15^ Abay reported that anemia was seen only in 46% of pulmonary tuberculosis (PTB) cases and PTB-HIV infected group showed increased frequency of anemia as seen in 60% cases.^16^ Mulenga reported anemia in 69% of cases and it was moderate in 92% of cases; iron deficiency anemia was predominant type and he showed that hypoalbuminemia, alcohol intake and C-reactive protein (CRP) had an effect on anemia.^17^ Kulkarni in 2017 also reported IDA as the predominant type and anemia was seen in 69.2% of cases and mostly it was mild.^18^

**Table-II:** Relationship of Age and Gender with Hematological abnormalities in newly diagnosed patients of pulmonary tuberculosis

Parameter	Anemia (Yes)	Leukocytosis (Yes)	Leucopenia (Yes)	Thrombocytosis (Yes)	Thrombocytopenia (Yes)	ESR Raised
***Age Groups (years)***						
20-29 (n=156)	130(26)	73(14.6)	28(5.6)	39(7.8)	18(3.6)	154(30.8)
30–39 (n=204)	165(33)	90(18)	37(7.4)	49(9.8)	17(3.4)	203(40.6)
> 39 (n=140)	118(23.6)	68(13.6)	37(7.4)	43(8.6)	18(3.6)	138(27.6)
p-value	0.686	0.634	0.614	0.351	0.367	0.631
***Gender***						
Male (n=358)	291(81.8)	163(83.59)	71(19.83)	82(22.91)	42(11.73)	354(98.88)
Female(n=142)	122(85.92)	68(47.89)	31(21.83)	49(34.51)	11(7.75)	141(99.29)
p-value	0.218	0.634	0.614	0.008*	0.367	0.675

* Significant.

Similarly, a study from central Punjab showed more anemia in co-morbid groups.^5^ Abay also supported this finding as he observed anemia more frequent in PTB-HIV co-infected groups as compared to PTB alone group.^16^ While in contrast to it, Hella reported that coinfections with HIV, helminths, and respiratory pathogens had no effect on anemia and he reported anemia of chronic disease was most common type and correlated with increased hepcidin levels.^19^ Barzegari concluded in a systematic review and meta-analysis that anemia of chronic disease was the predominant type seen in 49.82% cases, while iron deficiency anemia was seen in 20.17% cases; the prevalence of anemia was 61.53%, this finding was supported by Mishra.^20^,^21^

In our study leukocytosis was detected in 46.2% cases, leucopenia in 20.4% cases while 33.4% cases were having normal white cell count. Our finding is in accordance with Kulkarni, who reported leukocytosis in 38.4% of cases.^18^ Erhabor reported leukocytosis in only 21.25% of cases and most of the patients (76%) had normal white cell count. Leucopenia in PTB patients is due to immunosuppression and he also found a low frequency of leucopenia in his study, seen only in 3.75% of cases.^13^ Shafee reported leukopenia in 8% of males and 5% of females, while 60% of males and 64% of females were neutrophilic. Lymphopenia was seen in 59% of males and 43% of females, this finding was also supported by Panteleev.^12^,^22^

In our study, thrombocytopenia was seen in 10.6% of cases and thrombocytosis was seen in 26.2% of cases. Erhabor reported that thrombocytopenia was common in miliary tuberculosis and thrombocytosis was common in pulmonary tuberculosis. He did not find thrombocytopenia in any of his pulmonary TB patients, while 10% of cases reported thrombocytosis. Changes in the sedimentation rate exactly parallel alteration in the tuberculosis focus. Our study reported that 99% of patients had increased ESR. Kulkarni reported increased ESR in 49% cases,^18^ and Abay reported increased ESR in 100% cases.^16^ While Kahase noted deranged ESR in 85% cases a value lower than our study.^15^

Additionally, we stratified our data and found no association between age/gender and hematological complications. The only association was observed between gender and thrombocytosis with a higher proportion of male patients, p-value = 0.008 and it is in agreement to an Indian study by Mukherjee.^14^ Thrombocytosis could be a result of inflammation.^14^ But no previous studies in Pakistan showed this association.^5^,^6^,^12^ More studies are needed to confirm this trend of thrombocytosis in TB patients.

### Limitations:

It was a single-center, retrospective study that did not classify anemia, leukopenia and leukocytosis that can give more clue to hematological derangements occurring in tuberculosis patients before therapy and their measurement after therapy can show efficacy of therapy that is lacking in this study.

## CONCLUSION

Hematological parameters like high Erythrocyte Sedimentation Rate (ESR) and anemia were common hematological abnormalities detected in newly diagnosed patients with pulmonary tuberculosis. Thrombocytosis was seen more commonly in male patients of pulmonary tuberculosis.

### Authors’ Contribution:

**YB:** Study design, data collection, writing the manuscript, She is also responsible and accountable for the accuracy and integrity of the work.

**GP:** Data collection, Statistical analysis, interpretation of results.

**AA:** Drafting the article and revising it for important intellectual content. Statistical analysis, interpretation of results, **SF:** Study design, data collection, writing the manuscript, Final approval of version for publication.
